# Finger-Vein Image Enhancement Using a Fuzzy-Based Fusion Method with Gabor and Retinex Filtering

**DOI:** 10.3390/s140203095

**Published:** 2014-02-17

**Authors:** Kwang Yong Shin, Young Ho Park, Dat Tien Nguyen, Kang Ryoung Park

**Affiliations:** Division of Electronics and Electrical Engineering, Dongguk University, 26 Pil-dong 3-ga, Jung-gu, Seoul 100-715, Korea; E-Mails: skyandla@dongguk.edu (K.Y.S.); fdsarew@hanafos.com (Y.H.P.); datdtbkhn.nguyen@gmail.com (D.T.N.)

**Keywords:** finger-vein recognition, enhancement method, Gabor and Retinex filtering, fuzzy-based method

## Abstract

Because of the advantages of finger-vein recognition systems such as live detection and usage as bio-cryptography systems, they can be used to authenticate individual people. However, images of finger-vein patterns are typically unclear because of light scattering by the skin, optical blurring, and motion blurring, which can degrade the performance of finger-vein recognition systems. In response to these issues, a new enhancement method for finger-vein images is proposed. Our method is novel compared with previous approaches in four respects. First, the local and global features of the vein lines of an input image are amplified using Gabor filters in four directions and Retinex filtering, respectively. Second, the means and standard deviations in the local windows of the images produced after Gabor and Retinex filtering are used as inputs for the fuzzy rule and fuzzy membership function, respectively. Third, the optimal weights required to combine the two Gabor and Retinex filtered images are determined using a defuzzification method. Fourth, the use of a fuzzy-based method means that image enhancement does not require additional training data to determine the optimal weights. Experimental results using two finger-vein databases showed that the proposed method enhanced the accuracy of finger-vein recognition compared with previous methods.

## 1. Introduction

With the increased demand for personal information security, biometric technologies such as iris, face, fingerprint, finger-vein, voice, gait, palm-print, and hand geometry recognition have been employed in a wide number of security systems, e.g., building access, computer log-ins, door access control, cellular phones, and ATMs [1–4]. Biometric technology, which exploits the behavioral and/or physiological characteristics of an individual, has high distinctiveness, permanency, universality, usability, and performance capabilities [4]. In particular, finger-vein recognition systems are used to authenticate individuals as enrolled or non-enrolled, and it has various advantages, such as live detection and possible applications in bio-cryptography systems [5]. In human identification applications, finger-vein recognition uses the vein patterns detected inside the finger. When capturing a finger-vein image, the deoxyhemoglobin in the veins absorbs near infrared (NIR) light at a wavelength of 760–850 nm. The vein region in a finger-vein image thus appears as dark pixels, whereas the other regions appear as brighter pixels. Therefore, the area of a finger-vein image can be separated into regions with vein and non-vein patterns. The vein patterns of all fingers of the same person also have different characteristics. Therefore, to facilitate higher recognition accuracy, some finger-vein recognition systems use more than two fingers from the same individual.

Although finger-vein recognition is less affected by wounds or deformations on the finger than fingerprint recognition, finger-vein patterns can be ambiguous and unclear because of light scattering from the skin, low contrast, and uneven illumination. These factors degrade the quality of a finger-vein images and the discrimination of vein patterns, which reduces the accuracy of the finger-vein recognition. To overcome the performance degradation of finger-vein recognition, many previous studies have developed different enhancement methods for finger-vein images, some of which are compared with the proposed method in Table 1. Previous quality enhancement methods for finger-vein images can be classified into restoration-based and non-restoration-based methods [6].

For example, Yang *et al.* developed a restoration-based method that removes the optical blur from the camera lens and the skin scattering blur from the structure of the finger skin layers to transform a low-quality finger-vein image into a high-quality image [7]. They formulate the camera lens and skin scattering blurs by considering the optical characteristics of the skin layers using a Gaussian-based point spread function (PSF) model and a depth-PSF model. Several restored images are obtained based on various skin surface depth parameters because it is not possible to correctly estimate the depth of the skin surface in the vein region. In addition, a linear superposition method is employed to conjoin the several restored images to produce a combined image. However, this method is limited because the processing time is increased by obtaining several restored images with various skin surface depth parameters. To eliminate the skin scattering blur in a finger-vein image, an optical model based on skin scattering and atmospheric scattering components has also been used for enhancing finger-vein images [8]. This approach is based on de-hazing and the removal of skin scattering blur, which makes the vein patterns in a finger-vein image easier to distinguish. However, this method is limited because its performance can be affected by the detection of the scattering parameter. In addition, enhancement of the recognition accuracy was not discussed in this paper.

Yang *et al.* proposed an enhancement method for finger-vein images based on scattering removal, Gabor filtering, and a multi-scale multiplication rule [9]. However, they assumed that the luminance of the surrounding environment would be constant during processing to facilitate scattering removal. In addition, the optimal parameters of the Gabor filter were designed in an elaborate manner based on the characteristics of the vein lines. Therefore, the parameters need to be redesigned for vein images captured using different devices. By contrast, our proposed method uses a roughly designed Gabor filter, which has the advantage that its performance is not affected significantly by the different types of vein images (in this study, this was confirmed by tests using two finger-vein databases, which were collected with two different devices). In our method, performance enhancement is achieved using a combination of Gabor and Retinex filters based on a fuzzy system. The fuzzy system can be designed heuristically without a training procedure to obtain the optimal weights for the combination of Gabor and Retinex filtering. Therefore, this system has the advantage that it does not need to be redesigned for different finger-vein databases.

Depending on the number of images used, non-restoration-based methods can be divided into single image-based and multiple image-based enhancement methods. For example, Zhang *et al.* developed a single image-based approach [6,10–15], which uses gray-level grouping (GLG) for contrast enhancement and a circular Gabor filter (CGF) for image enhancement to increase the quality of finger-vein images [10]. Pi *et al.* introduced a quality improvement approach based on edge preserving and elliptical high pass filters to maintain the edges and remove any blur [11]. Histogram equalization is then used to increase the contrast of the resulting image. In addition, a fuzzy-based multi-threshold algorithm, which considers the characteristics of the vein patterns and the skin region, was proposed by Yu *et al.* [12]. This fuzzy-based multi-threshold algorithm is not only straightforward, but it also increases the contrast between the vein patterns and the background. Yang *et al.* introduced an enhancement method that uses multi-channel even-symmetric Gabor filters with four directions and three center frequencies to obtain distinct vein patterns [13]. After obtaining the filtered images, an enhanced image is generated by combining the filtered images based on a reconstruction rule. However, enhanced recognition accuracy was not demonstrated in any of these previous studies [10–13].

Park *et al.* proposed an image quality enhancement method that considers the direction and thickness of the vein line based on an optimal Gabor filter [6], where they determine the direction of the vein lines based on eight directional profiles of a gray image and the thickness of the vein lines based on the optimal Gabor filter width. This method improves the visibility of the resulting finger-vein image and the recognition accuracy using the enhanced images. However, this method uses two-step Gabor filtering (four directional Gabor filters and optimal Gabor filtering based on eight directions), which increases the processing time. In addition, detection errors in the orientation and thickness of the vein line can affect the performance. Yang *et al.* introduced a line filter transform (LFT) to compute the primary orientation field (POF) of a finger-vein image after using the curvatures of the cross-sectional profiles to estimate the coarse vein-width variation field (CVWVF) [14]. The venous regions are enhanced by the curve filter transform (CFT), and the visibilities of the vein region and vein ridges are clearly improved. However, detection errors in the orientation and thickness of a vein line could affect the performance. To enhance the quality of a finger-vein image, Cho *et al.* presented an adaptive Gabor filtering method based on the orientation and width of a detected finger-vein line [15], where a finger-vein line detected using vein line tracking is used to measure the orientation of the finger-vein.

The width of a finger-vein is obtained using the gray profiling of the original image that corresponds to the finger-vein line. However, the image enhancement performance could be degraded by inaccurate detection of the vein orientation and width in all previous studies [6,14,15]. Kumar *et al.* proposed a system that combined finger-vein and fingerprint recognition results using a novel score-level fusion method [16]. The finger-vein image is enhanced based on the average background image and local histogram equalization. However, skin areas become uneven with this method, despite the distinctiveness of the vein line. Thus, vein line detection is required based on further processing by matched filtering, repeated line tracking, maximum curvature detection, Gabor filtering, and morphological operations. However, our method has the advantage that the image produced after image enhancement can be used for recognition without further processing.

In [17], the authors proposed a quality assessment method for finger-vein images, but they did not consider quality enhancement. Miura *et al.* proposed a robust method for extracting the centerlines of veins by calculating the local maximum curvatures in cross-sectional profiles of vein images [18]. However, this study aimed to locate an accurate vein line and it did not focus on vein image enhancement, which differs from our method for enhancing finger-vein images. Yang *et al.* proposed a method for evaluating the finger-vein image quality using a trained support vector machine (SVM), which was based on the gradient, image contrast, and information capacity of the image [19]. However, this method was used for quality evaluation rather than finger-vein image enhancement, which differs from our method for enhancing the finger-vein image.

Nguyen *et al.* [20] proposed a method for detecting fake finger-vein images, which combined the features of the Fourier transform, and Haar and Daubechies wavelet transforms based on a SVM. However, their method was used for detecting fake finger-vein images rather than finger-vein image enhancement, which differs from our method.

In this study, we propose a novel finger-vein image enhancement method to overcome the problems of previous methods. Four directional Gabor filters and Retinex filtering are used to amplify the local and global features of the vein lines in an input image. The two images produced by Gabor and Retinex filtering are combined to obtain an enhanced image based on a fuzzy-based fusion method. Gabor and Retinex filtering are both common image enhancement methods, but the main novelty of our approach is the fuzzy-based combination method for Gabor and Retinex filtering. The fuzzy system can be designed heuristically without a training procedure to obtain the optimal weights for the combination of Gabor and Retinex filtering. Therefore, this system has the advantage that it does not need to be redesigned for different finger-vein databases whereas a neural network-based system must be trained to suit specific databases. The means and standard deviations in the local windows of the images produced after Gabor and Retinex filtering are used as the inputs for the fuzzy rule and fuzzy membership function, respectively. The optimal weights used to combine the Gabor and Retinex filtered images are determined using a defuzzification method.

The remainder of this paper is organized as follows: in Section 2, the proposed method is described, including the detection of a finger-vein region, Gabor filtering in four directions, Retinex filtering, the proposed image-fusion method based on fuzzy theory, and a finger-vein recognition method. The experimental results and some concluding remarks are given in Sections 3 and 4, respectively.

## 2. Proposed Fuzzy-Based Fusion Method for Finger-Vein Image Quality Enhancement

### 2.1. Overview of the Proposed Approach

**Figure 1.**
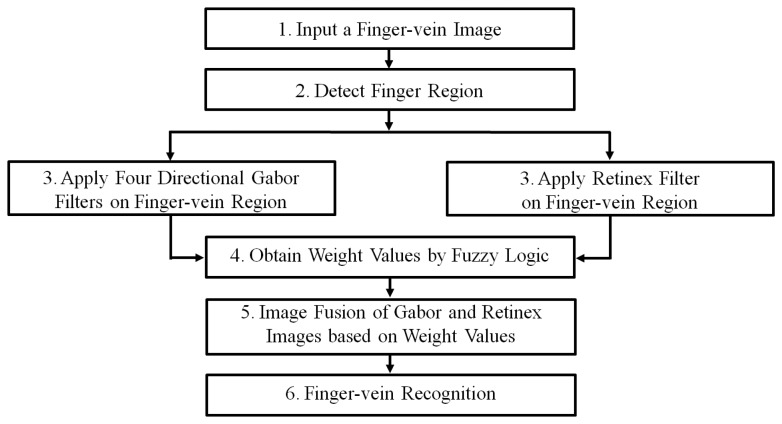
Flowchart showing the proposed fuzzy-based fusion method for finger-vein image enhancement.

Figure 1 shows a flowchart of the proposed fuzzy-based fusion method for finger-vein image enhancement. After inputting a finger-vein image, the finger-vein region is detected using detection masks that are applied to the upper and lower finger boundaries [6,17,20], which eliminates the allocation of unnecessary processing time to an enhancement procedure for the background region (see Step 2 in Figure 1 and Section 2.2). To amplify the local and global features of the vein lines in an input image, Gabor filtering in four directions and Retinex filtering are employed to generate two images (see Step 3 in Figure 1 and Sections 2.3 and 2.4). The optimal weight values for combining the Gabor and Retinex images are obtained using a fuzzy rule, fuzzy membership function, and defuzzification method based on the means and standard deviations (STDs) measured in the local windows of the two resulting images (see Step 4 in Figure 1 and Section 2.5). The Gabor and Retinex images are combined using the determined optimal weights (see Step 5 in Figure 1 and Section 2.5). Finger-vein recognition is conducted using the combined image, including size normalization based on stretching and sub-sampling, feature extraction, and code matching to identify whether the subject is genuine or an imposter (see Step 6 in Figure 1 and Section 2.6).

### 2.2. Finger Region Detection

**Figure 2.**
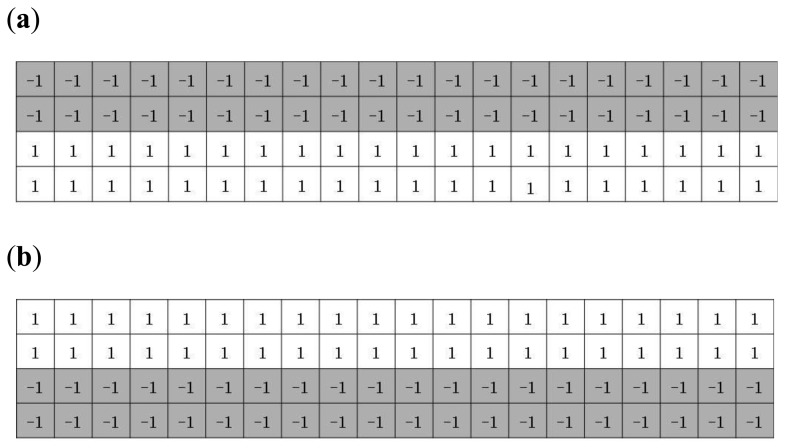
Detection masks for the (**a**) upper and (**b**) lower finger boundaries [6,17,20].

A captured finger-vein image is separated into finger-vein and background regions because the latter do not contain the vein patterns used for finger-vein recognition. The background region appears as dark pixels whereas the finger-vein region appears as bright pixels, which means that detection masks over the upper and lower finger boundaries can be used to find the finger-vein region, as shown in Figure 2 [6,17,20]. The mask size was determined empirically as 20 × 4 pixels.

**Figure 3.**
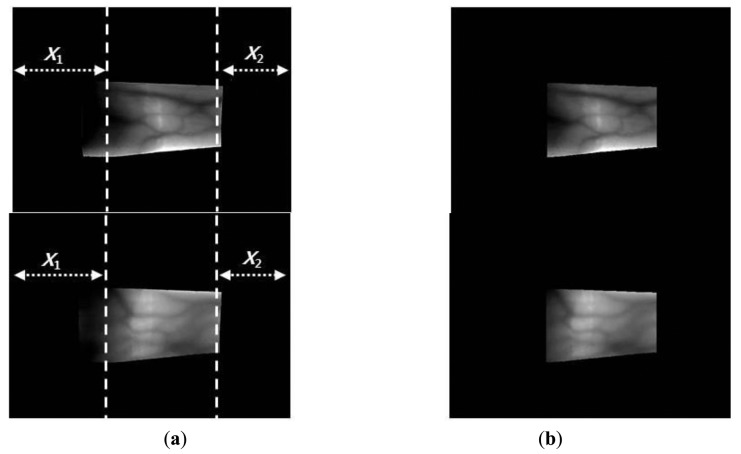
Examples of finger region detection using images from database I: (**a**) original images and (**b**) detection results for the finger boundaries.

**Figure 4.**
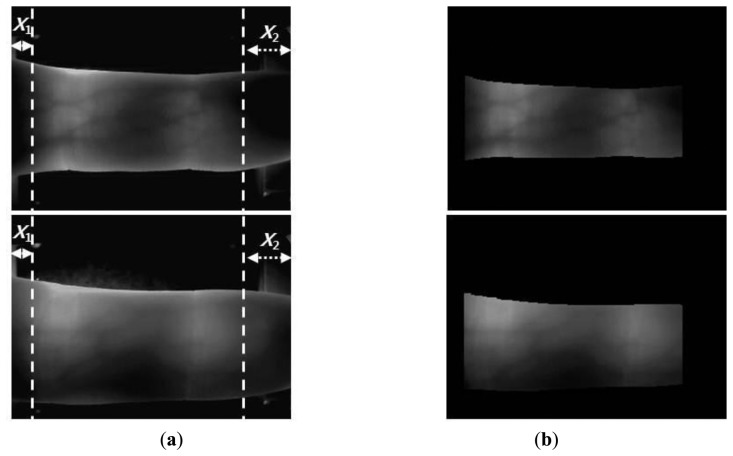
Examples of finger region detection using images from database II: (**a**) original images and (**b**) detection results for the finger boundaries.

The y-positions indicate where the maximum values for template matching are obtained (at each x-position) using the detection masks shown in Figure 2, which are considered the upper and lower edge boundaries [6,17,20]. A thick finger area (for example, the left part of Figure 3a) usually lacks a vein pattern because the NIR light has difficulty penetrating the thick finger to be captured by the camera. In addition, the thin vein pattern information is not visible in the fingertip region of a captured finger-vein image. To consider these conditions, we define the left (*X_1_*) and right (*X_2_*) boundaries in the horizontal direction, as shown in Figures 3a and 4a. The values of *X_1_* and *X_2_* were defined empirically based on the characteristics of the finger-vein database used in the experiments. In database I, with a 640 × 480 image size [6,17,20], the values of *X_1_* and *X_2_* are 220 and 169, respectively. In database II, with a 320 × 240 image size [21], the values of *X_1_* and *X_2_* are set to 20 and 51, respectively (detailed explanations of databases I and II are provided in Section 3). The values of *X_1_* and *X_2_* are larger in database I than those in database II for the following reasons. Database I was collected using a device produced in our laboratory. The device includes a hole where the finger that needs to be recognized is placed. The hole is small but a small area of the finger, *i.e.*, the region between the 1st and 2nd knuckles, can be observed through the hole by the camera in the device. Therefore, the unseen (dark) areas from the left and right boundaries of the image are larger in database I than those in the database II, as shown in Figures 3 and 4. Consequently, we used the larger values for *X_1_* and *X_2_* in database I. Figures 3 and 4 show examples of finger region detection using detection masks. Because of the noise in the upper and lower boundaries of the finger region of database II, the region of interest used for finger-vein recognition is reduced in the vertical direction compared with the detected finger region, as shown in Figure 4.

### 2.3. Finger-Vein Image Enhancement Method Based on Four-Directional Gabor Filtering Algorithm

In general, a two-dimensional (2D) Gabor filter is defined as a Gaussian function that comprises a complex sinusoidal signal. The Gaussian function can be expressed as Equations (1) and (2) [6,13,22]:

(1) $$G(x,y)=\frac{1}{2\pi \sigma_{x}\sigma_{y}}\exp\left\{-\frac{1}{2}\left(\frac{x_{\theta }^{2}}{\sigma_{x}^{2}}+\frac{y_{\theta }^{2}}{\sigma_{y}^{2}}\right)\right\}\exp(\hat{j}2\pi f_{0}x_{\theta })$$

where:

(2) $$\left[\begin{array}{c} x_{\theta }\\ y_{\theta } \end{array}\right]=\left[\begin{array}{cc} \cos\theta & \sin\theta \\ -\sin\theta & \cos\theta \end{array}\right]\;\left[\begin{array}{c} x\\ y \end{array}\right]$$

The parameters *σ_x_* and *σ_y_* determine the space-domain envelope of the Gaussian function on the x- and y-coordinates in 2D [6,13,22], respectively, *x_θ_* and *y_θ_* indicate the rotated x- and y-coordinates of a 2D Gabor filter based on a *θ* rotation rate, respectively [6,13,22], and *ĵ* and *f_0_* represent $\sqrt{-1}$ and the spatial center frequency of the filter, respectively [6,13,22]. In this method, the real part of the Gabor filter is employed only to increase the effectiveness of the processing time by eliminating the imaginary part of the Gabor filter. An even-symmetric Gabor filter without an imaginary part can be expressed as [6,13,22]:

(3) $$G_{i}^{e}(x,y)=\frac{1}{2\pi \sigma_{x}\sigma_{y}}\exp\left\{-\frac{1}{2}\left(\frac{x_{\theta_{i}}^{2}}{\sigma_{x}^{2}}+\frac{y_{\theta_{i}}^{2}}{\sigma_{y}^{2}}\right)\right\}\cos(2\pi f_{i}x_{\theta_{i}})$$

where *i* (*i* = 1,2,3,4) indicates the channel index in four directions, *θ_i_*(=*iπ*/*4*) is the orientation of the *i^th^* channel of the Gabor filter, and *f_i_* represents the spatial center frequency of the even-symmetric Gabor filter according to the *i^th^* channel. *f_i_*, *σ_x_*, and *σ_y_* are 0.05, 9.53, and 9.53, respectively.

**Figure 5.**
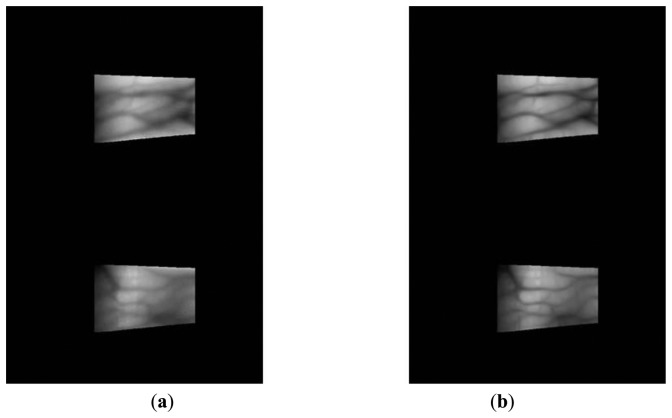
Examples of the results obtained by four-directional Gabor filtering using images from database I: (**a**) the original images with the detected finger boundaries and (**b**) the images produced after the application of filtering.

**Figure 6.**
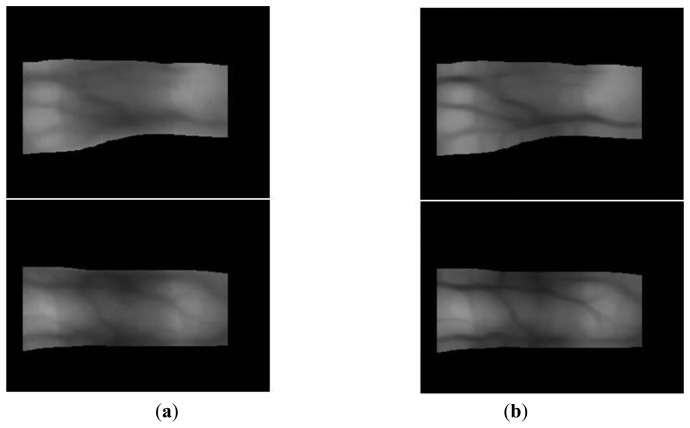
Examples of the results obtained by four-directional Gabor filtering using images from database II: (**a**) the original images with the detected finger boundaries; and (**b**) the images produced after the application of filtering.

As shown in Figures 3 and 4, the finger-veins follow various directions, such as horizontal, vertical, and diagonal. Therefore, we use four Gabor filters in four directions, *i.e.*, 45°, 90°, 135°, and 180°, as shown in Equation (3), to increase the amplitudes of the vein lines in various directions. For each channel, the filtered image (*O_i_*(*x_i_*,*y_i_*)) is obtained by convoluting the original image (I(*x*,*y*)) using the corresponding Gabor kernel $\left(G_{i}^{e}(x,y)\right)$, as follows [6,13,22]:

(4) $$O_{i}(x,y)=G_{i}^{e}(x,y)*I(x,y)$$

where * is the convolution operator. Using Equations (3) and (4), the four resulting images are generated according to the four directions (−45°, 0°, 45°, and 90°) of the Gabor filter. To combine the four resulting images and produce the final image, the lowest gray-level value among the four resulting images at the same position is chosen as the best match for the Gabor filter because the finger-vein line is darker than the skin region [6,13]. Figures 5 and 6 show images produced using four directional Gabor filtering for database I and database II, respectively. As shown in these figures, the finger-vein lines are more distinct than those in the original image.

### 2.4. Finger-Vein Image Enhancement Using Retinex Filtering Algorithm

To enhance the distinctiveness of the image, the Retinex algorithm is introduced by reducing the variance in the image illumination to normalize the image illumination [23,24]. The intensity of the captured image (*L*(*x*, *y*)) is modeled by multiplying the illumination (*I_c_*(*x*, *y*)) and the ratio of reflection (*r*(*x*, *y*)) [24]:

(5) $$L(x,y)=I_{c}(x,y)\times r(x,y)$$

From Equation (5), we can obtain Equation (6) [23]:

(6) $$\log r(x,y)=\log L(x,y)-\log I_{c}(x,y)$$

**Figure 7.**
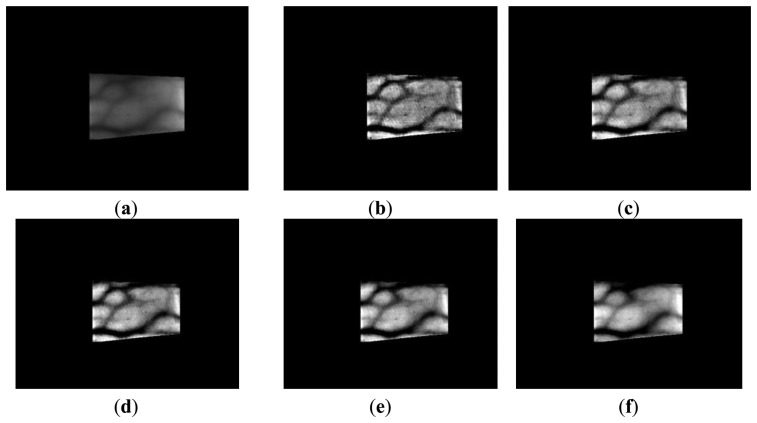
Retinex images obtained using various sigma values with images from database I: (**a**) the original image with the detected finger boundaries; Retinex images obtained using sigma values of (**b**) 10, (**c**) 15, (**d**) 20, (**e**) 25, and (**f**) 50.

**Figure 8.**
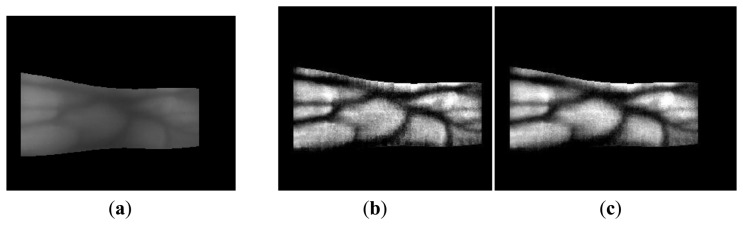

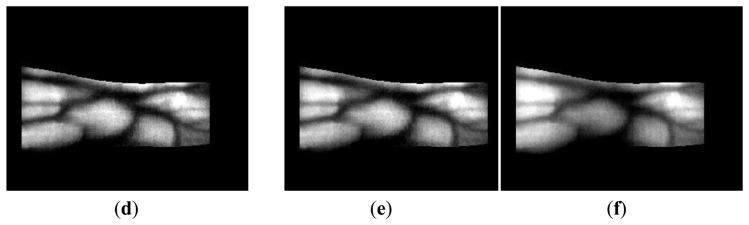
Retinex images obtained using various sigma values with images from database II: (**a**) the original image with the detected finger boundaries; Retinex images obtained using sigma values of (**b**) 10, (**c**) 15, (**d**) 20, (**e**) 25, and (**f**) 50.

The illumination (*I_c_*(*x*, *y*)) is assumed to be a convolution of the Gaussian filtering (*F*(*x*, *y*)) and the image(*L*(*x*, *y*)), as shown in Equations (7) and (8) [24]:

(7) logr(x,y)=logL(x,y)−log[L(x,y)*F(x,y)]

(8) $$F(x,y)=\frac{1}{2\pi \sigma^{2}}e^{-\frac{x^{2}+y^{2}}{2\sigma^{2}}}$$

where *F*(*x*,*y*) and log*r*(*x*, *y*) indicate the Gaussian filter and the image produced after Retinex filtering. Retinex images obtained using various sigma values (*σ* = 10, 15, 20, 25, 50) for Gaussian filtering are shown in Figures 7 and 8. The vein patterns in the images produced after Retinex filtering are more distinct, and the contrast between the vein patterns and the skin regions is higher than that in the original images.

### 2.5. Finger-Vein Image Enhancement Method with a Fuzzy-based Fusion Approach

**Figure 9.**
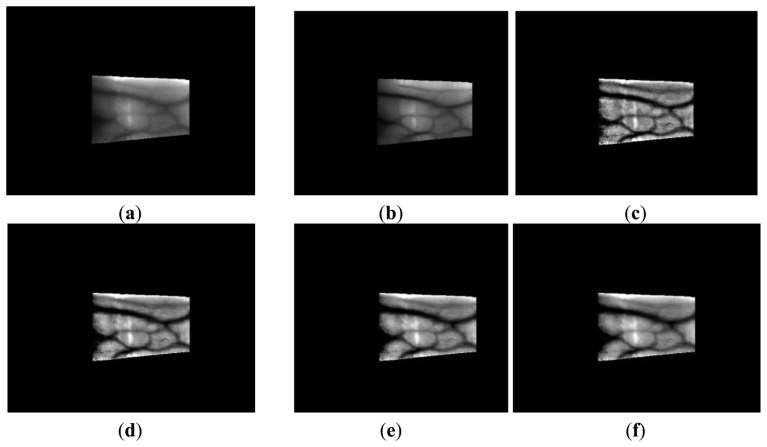

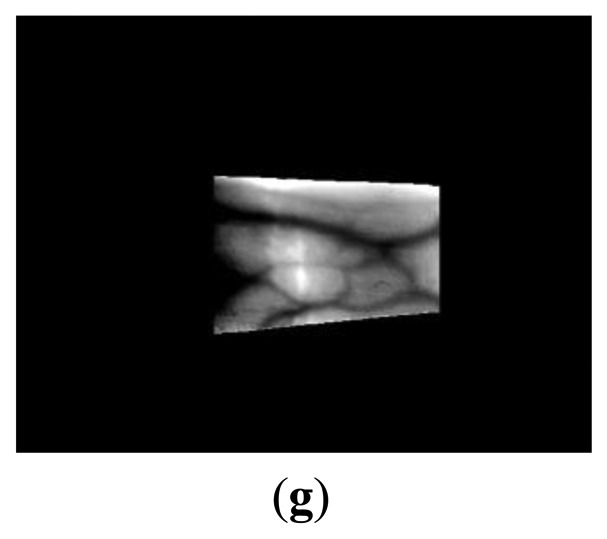
Comparison of outputs produced by Gabor and Retinex filtering using images from database I: (**a**) original image of the detected finger boundaries; (**b**) results with Gabor filtering; and results with Retinex filtering using sigma values of (**c**) 10, (**d**) 15, (**e**) 20, (**f**) 25, and (**g**) 50.

**Figure 10.**
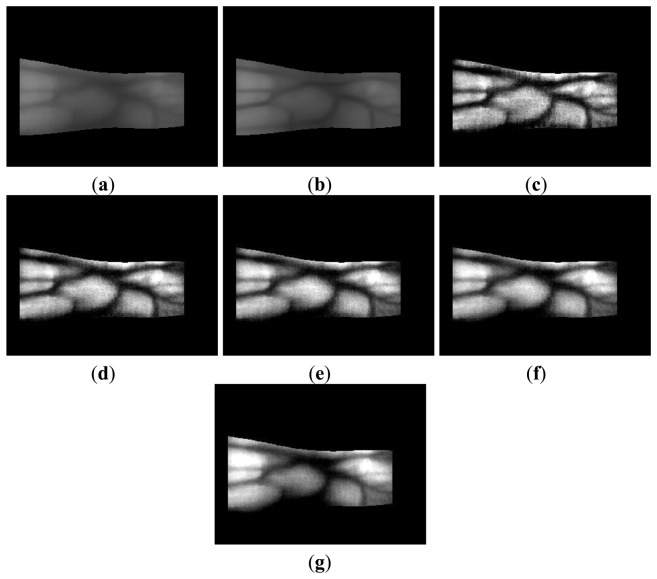
Comparison of the outputs with Gabor and Retinex filtering using images from database II: (**a**) original image of the detected finger boundaries; (**b**) results with Gabor filtering; and results with Retinex filtering using sigma values of (**c**) 10, (**d**) 15, (**e**) 20, (**f**) 25, and (**g**) 50.

The enhancement of thick vein lines is limited by the four-directional Gabor filter, whereas the thin vein lines become more distinct, as shown in Figures 9b and 10b. However, the thick vein lines are more distinct with Retinex filtering, as shown in Figures 9 and 10. Therefore, we can estimate that the local and global features of the vein lines are enhanced by the Gabor and Retinex filters, respectively. To enhance both the local and global features, we propose a fuzzy-based image fusion method for combining the Gabor and Retinex filtered images.

#### 2.5.1. Definition of the Membership Function

**Figure 11.**
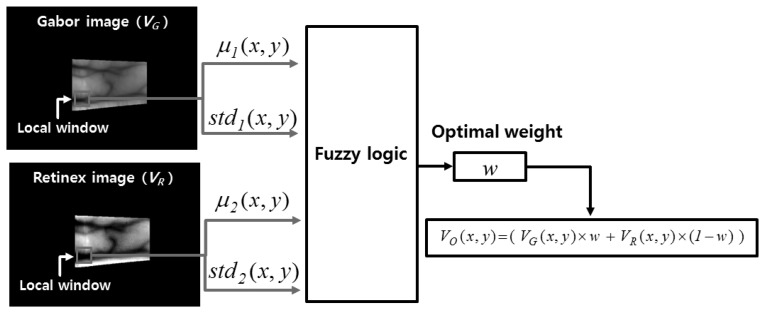
Illustration of the proposed fuzzy-based image fusion method.

Figure 11 illustrates the proposed fuzzy-based image fusion method. The mean (*μ*(*x*,*y*)) and STD (*std*(*x*,*y*)) values in the local windows of the images produced by Gabor and Retinex filtering are used as the inputs for the fuzzy logic system, as shown in Figure 11. We apply the local window by overlapping with a 1-pixel step. *μ_1_*(*x*, *y*), *std_1_*(*x*, *y*), *μ_2_*(*x*, *y*), and *std_2_*(*x*, *y*) (which are normalized based on a min-max scale of 0 to 1) denote the mean and STD values measured in the local window according to the center position (*x*, *y*) of the Gabor and Retinex images, respectively; *V_G_*(*x*, *y*) and *V_R_*(*x*, *y*) are the pixel values of the Gabor and Retinex filtered images at the (*x*, *y*) position, respectively; *w* and *V_O_*(*x*, *y*) indicate the optimal weight values obtained using fuzzy logic and the pixel value of the enhanced combined image obtained from the Gabor and Retinex images at the (*x*, *y*) position, respectively. The horizontal and vertical lengths of the local square window are set as two times larger than the greatest width of the vein lines in the Gabor and Retinex filtered images.

The STD value in a local window that includes a vein line is usually larger than that of a window that includes only the skin area. In addition, a vein line is included in the local window and the mean value of the local window is lower because the vein line is darker than the skin area. Therefore, we can obtain the following relationships. If the mean and STD values in the local window are low and high, respectively, the possibility that the local window contains a vein line is high. By contrast, if the mean and STD values in the local window are high and low, respectively, the possibility that the local window contains a vein line is low. Based on this relationship, we determine the fuzzy rules, which have four inputs, *i.e.*, the mean (*μ_1_*(*x*, *y*)) and STD (*std_1_*(*x*, *y*)) for the Gabor filtered image, and the mean (*μ_2_*(*x*, *y*)) and STD (*std_2_*(*x*, *y*)) for the Retinex filtered image, in the local window, as shown in Table 2. A detailed explanation of Table 2 is given in Section 2.5.2.

**Figure 12.**
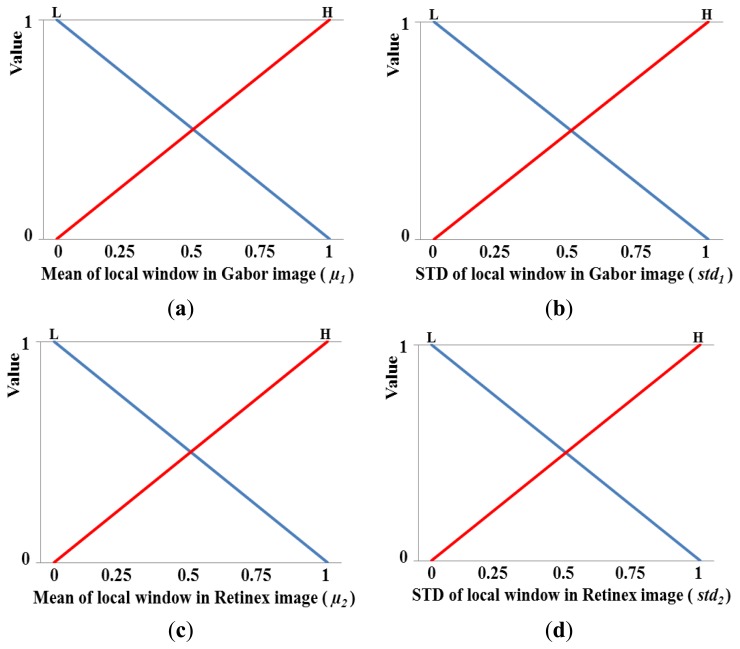

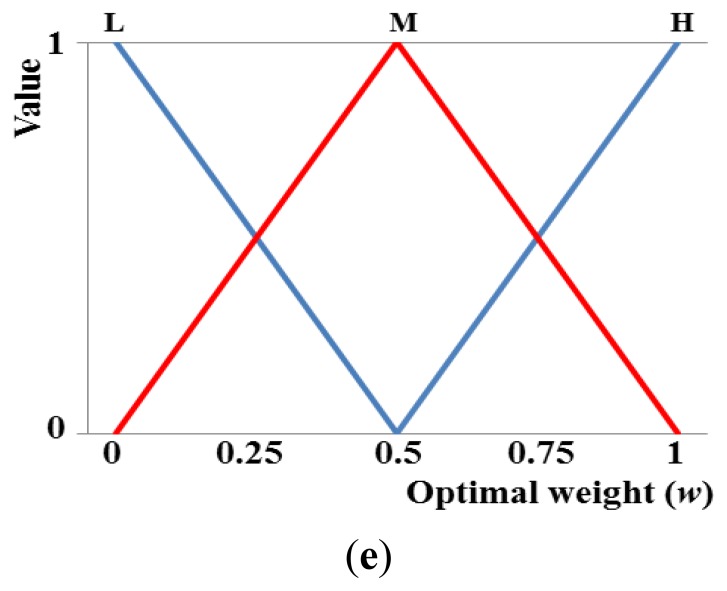
Membership functions used for fuzzy-based image fusion: (**a**) mean and (**b**) standard deviation (STD) of the local window in a Gabor filtered image; (**c**) mean and (**d**) STD of the local window in a Retinex filtered image; and (**e**) membership function for obtaining the optimal weight.

Figure 12a–d shows the membership functions for the input values. As shown in these figures, each of the four inputs is categorized as low (L) and high (H) based on a linear membership function. A membership function usually represents the distributions of the input or output values for a fuzzy system. Thus, we define the two distributions (L and H) for the mean in the local window of the Gabor image shown in Figure 12a. In general, two distributions can share some common areas, and therefore, we define the two distributions of L and H that include an overlapping region, as shown in Figure 12a. The number of input values is as high as four in Figure 12a–d, and therefore, we only use two membership functions (L and H) for each input value to reduce the number of fuzzy rules in Table 2. However, the optimal output weight needs to be represented in detail; therefore, we use three membership functions for L, M, and H, as shown in Figure 12e. Consequently, the optimal weight (*w*) of the fuzzy output used to combine the Gabor and Retinex images is obtained using the membership function for the output value, as shown in Figure 12e.

#### 2.5.2. Fuzzy Rules that Consider the Characteristics of Gabor and Retinex Images

As described in Section 2.5.1, if the mean and STD values in the local window are low (L) and high (H), respectively, the possibility that the local window contains a vein line is high (H). Conversely, if the mean and STD values in the local window are high (H) and low (L), respectively, the possibility that the local window contains a vein line is low (L). Based on these relationships, 16 types of fuzzy rules are determined using four (L) or (H) inputs to obtain the optimum weighting value required for image fusion, as shown in Table 2. The weighting value of a Retinex filtered image is determined as 1-*w*, as shown in Figure 11. As shown in Table 2, if *u_1_* and *std_1_* for a Gabor filtered image are L and H, respectively, and *u_2_* and *std_2_* for a Retinex filtered image are H and L, respectively, we assign the larger weighting value (H) to the Gabor filtered image because the possibility that the local window of this image contains a vein line is larger. If *u_1_* and *std_1_* for the Gabor filtered image, and *u_2_* and *std_2_* for the Retinex filtered image are L or H, we assign the same weighting value (M) to the Gabor and Retinex filtered images, respectively, because it is difficult to determine the local windows of the Gabor and Retinex filtered images that has a higher possibility of containing a vein line.

For a high (H) *μ_1_*, high (H) *std_1_*, high (H) *μ_2_*, and low (L) *std_2_*, although the high (H) mean value of the local window indicates that this window region contains more skin area than vein lines, we assign the larger weighting value (H) to the Gabor filtered image because the STD value of the local window of this image is higher than that of the Retinex filtered image (the possibility that the local window of the Gabor filtered image contains a vein line is larger).

#### 2.5.3. Determination of the Optimal Weights Using Defuzzification

**Figure 13.**
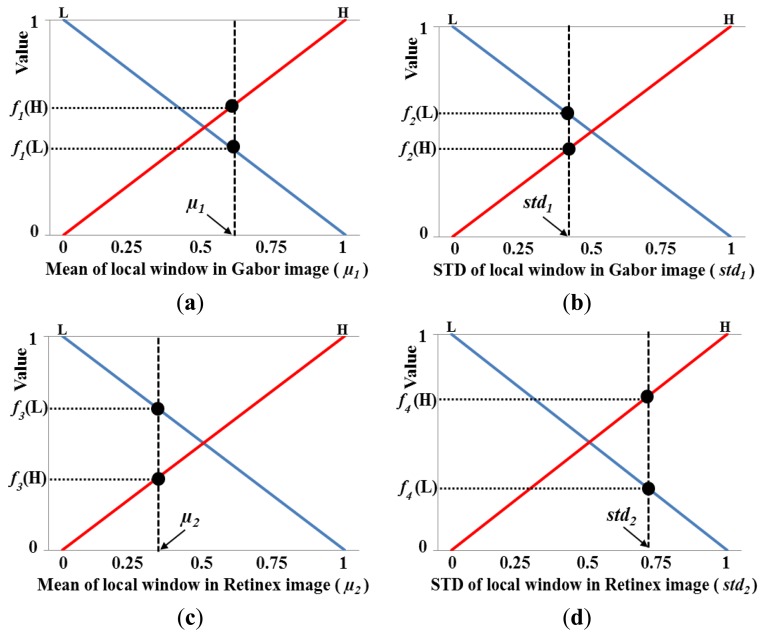
Illustrations showing the linear membership outputs based on four input values: (**a**) *μ_1_*, (**b**) *std_1_*, (**c**) *μ_2_*, and (**d**) *std_2_*.

Using the four input values (*μ_1_*, *std_1_*, *μ_2_*, and *std_2_*) obtained in the local window, the eight corresponding output values are calculated as *f_1_*(L) and *f_1_*(H) for *μ_1_*, *f_2_*(L) and *f_2_*(H) for *std_1_*, *f_3_*(L) and *f_3_*(H) for *μ_2_*, and *f_4_* (L) and *f_4_* (H) for *std_2_* using four linear membership functions, as shown in Figure 13, where, *f_1_*(˙), *f_2_*(˙), *f_3_*(˙), and *f_4_*(˙) are the membership functions that correspond to *μ_1_*, *std_1_*, *μ_2_*, and *std_2_*, respectively. Therefore, 16 combination pairs of the above output values are obtained as {(*f_1_*(L), *f_2_*(L), *f_3_*(L), *f_4_* (L)), (*f_1_*(L), *f_2_*(L), *f_3_*(L), *f_4_* (H)), (*f_1_*(L), *f_2_*(L), *f_3_*(H), *f_4_* (L)), (*f_1_*(L), *f_2_*(L), *f_3_*(H), *f_4_* (H)),…(*f_1_*(H), *f_2_*(H), *f_3_*(H), *f_4_* (H))}. Assuming that the values of *f_1_*(L), *f_1_*(H), *f_2_*(L), *f_2_*(H), *f_3_*(L), *f_3_*(H), *f_4_*(L), and *f_4_*(H) are 0.39, 0.61, 0.55, 0.45, 0.67, 0.33, 0.27, and 0.73, respectively, we can obtain the values listed in Table 3 based on the values in Table 2.

In general, the Min and Max rules are used to determine the deduced value from a combination pair [24,25]. Therefore, we can choose the minimum and maximum of the four values in a combination pair using the Min and Max rules, respectively. For example, if the four values of a combination pair are 0.39(L), 0.55(L), 0.67(L), and 0.27(L), the values of 0.27 and 0.67 are selected using Min and Max rules, respectively. In addition, if all four values are L, the corresponding output is M, as shown in the fuzzy rules defined in Table 2. Consequently, the values of 0.27(M) and 0.67(M) are selected using Min and Max rules, respectively, with the fuzzy rules in Table 2. Thus, the 16 types of deduced values based on the Min and Max rules, and the values listed in Table 2 are determined in this manner. In the present study, the deduced value is called the inference value (IV) for convenience [24].

**Figure 14.**
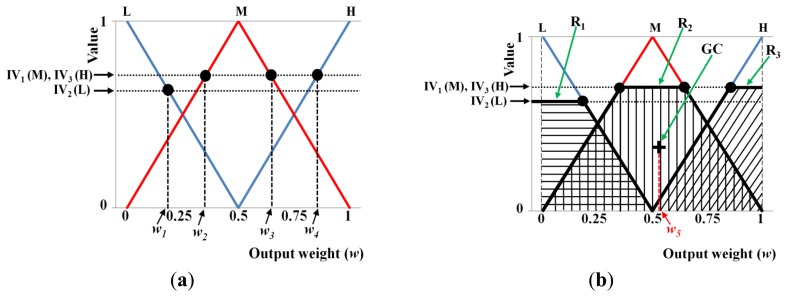
Illustration of the defuzzification methods used: (**a**) FOM, LOM, MOM, and MeOM; and (**b**) COG.

Using these 16 IVs, we can obtain the final optimal weightings based on the defuzzification step. Figure 14 shows an example of defuzzification using the IVs and the membership function for the output value (weight). With each IV, we can obtain the output values (*w_1_*, *w_2_*, *w_3_*, *w_4_*, and *w_5_* in Figure 14). Various defuzzification operators are introduced, *i.e.*, the first of maxima (FOM), last of maxima (LOM), middle of maxima (MOM), mean of maxima (MeOM), and center of gravity (COG) [24,26]. In Figure 14a, the FOM method selects the minimum value (*w_2_*) among the weight values calculated using the maximum IV (IV_1_(M) and IV_3_(H)) as the output weight. The LOM method selects the maximum value (*w_4_*) among the weight values calculated using the maximum IV (IV_1_(M) and IV_3_(H)) as the output weight. The MOM method selects the middle value ((*w_2_* + *w_4_*)/2) among the weight values calculated using the maximum IV (IV_1_(M) and IV_3_(H)) as the output weight. Finally, the MeOM method selects the mean value ((*w_2_* + *w_3_* + *w_4_*)/3) among the weight values calculated using the maximum IV (IV_1_(M) and IV_3_(H)) as the output weight. The output (score) calculated by the COG is *w_5_*, as shown in Figure 14b, which is the geometrical center (GC) of the union area of three regions (R_1_, R_2_, and R_3_). Using various defuzzification methods, the output weights are determined for the Gabor filtered image (*w* in Figure 11) and for the Retinex filtered image (1-*w* of Figure 11).

Table 2 shows that the number of fuzzy rules is 16 (2 × 2 × 2 × 2). If we use three distributions of L, M, and H as the input membership function, the number of fuzzy rules becomes 81 (3 × 3 × 3 × 3), which is considerably high and complex for use in a fuzzy system. Therefore, we simply use an input membership function based on the two distributions of L and H. However, the three distributions of L, M, and H are used as the output membership function to obtain more detailed values for the fuzzy system output.

### 2.6. Finger-Vein Recognition Method

**Figure 15.**
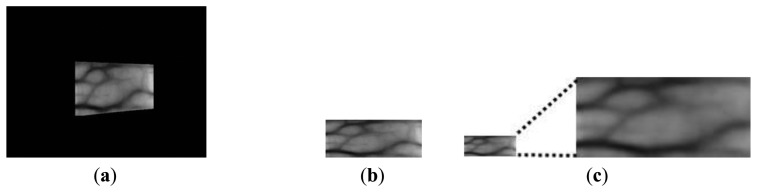
Images obtained using size normalization and downsampling: (**a**) original image with the detected finger boundaries; (**b**) a rectangular 150 × 60 pixel image using linear stretching based on the detected finger boundaries; and (**c**) a downsampled 50 × 20 pixel image.

Finger-vein recognition is performed after obtaining the enhanced image using the fuzzy-based fusion method, including size normalization, code extraction, and code matching [6,17,20]. Size normalization using linear stretching based on the detected finger boundaries (see Step 2 in Figure 1 and Section 2.2) is performed to reduce the variations in the shape and size of each finger. The finger-vein image is transformed into a rectangular 150 × 60 pixel image, as shown in Figure 15b [6,17,20]. This rectangular image is then downsampled to a 50 × 20 pixel image by taking the average gray-level value in each 3 × 3 pixel sub-block to enhance the processing speed for code extraction and matching, as shown in Figure 15c [6,17,20].

**Figure 16.**
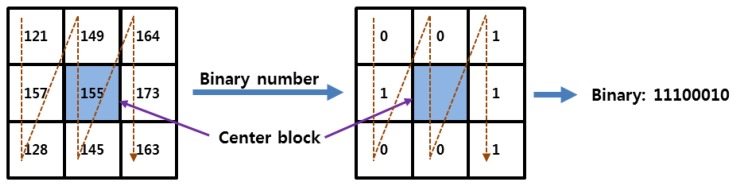
Example of finger-vein code extraction using a LBP operator.

Various feature extraction methods, such as the local binary pattern (LBP) and discrete wavelet transform (DWT) based on Daubechies and Haar wavelets [6,20], are used to evaluate the performance of the proposed finger-vein image enhancement method. First, the binary codes of the local features in the finger-vein image are extracted using an LBP operator [6,20]. Figure 16 shows an example of 8-bit binary code extraction using the LBP method. Because 8-binary bits are produced for each pixel position ((*x_c_*, *y_c_*)), 6,912-bit binary codes (8 (bits) × 48 (width) × 18 (height)) are generated by the LBP operator using a single down-sampled 50 × 20 pixel image.

The Hamming distance (HD) is used to obtain the matching score (distance) between enrolled and input binary codes using an LBP operator, as shown in Equation (9) [6,17,20]:

(9) $$HD=\frac{1}{N}(\text{BCE}⊕\text{BCI})$$

where *BCE* and *BCI* denote the enrolled and input binary codes, respectively, and ⊕ and *N* represent the Boolean exclusive OR operator and the total number of bits (6,912) of the binary codes, respectively. During iris recognition, non-iris areas such as eyelashes and eyelids are generally not used for recognition. The iris codes extracted from the non-iris areas are designated as invalid codes and they are not used to calculate the HD. However, all of the texture areas, including finger-veins and skin regions of the finger, are used for matching in our method. Therefore, a scheme that only uses valid codes is not adopted in Equation (9).

The input image is decomposed into four sub-band regions (LL, LH, HL, and HH) using Daubechies and Haar wavelets. The global features used for finger-vein recognition are extracted from these regions [6,20,27]: the LL and HH sub-bands are characterized as low- and high-frequency components, respectively, according to the vertical and horizontal directions; the LH sub-band is characterized as a conjoined low-frequency component in the vertical direction by a scaling function and a high-frequency component in the horizontal direction by a wavelet function; the HL sub-band is characterized as a conjoined high-frequency component in the vertical direction and a low-frequency component in the horizontal direction by wavelet and scaling functions, respectively [6,27]. Using the DWT with three-level decomposition, 64 sub-space regions are obtained, and the mean and STD values in each sub-space region are extracted as global features. From the DWT image, 128 features (2 (mean and STD in each sub-space region) × 64 (sub-space regions)) are obtained, which are normalized by min-max scaling. To determine the matching score based on the global features of the enrolled and input finger-vein images, the matching score of the Euclidean distance (ED) can be calculated as follows:

(10) $$ED=\frac{1}{M}\sqrt{\sum_{i=1}^{M}{\left(p_{i}-q_{i}\right)}^{2}}$$

where *p_i_*, *q_i_*, and *M* indicate the enrolled and input global features, and the number of global features (128), respectively.

## 3. Experimental Results

**Figure 17.**
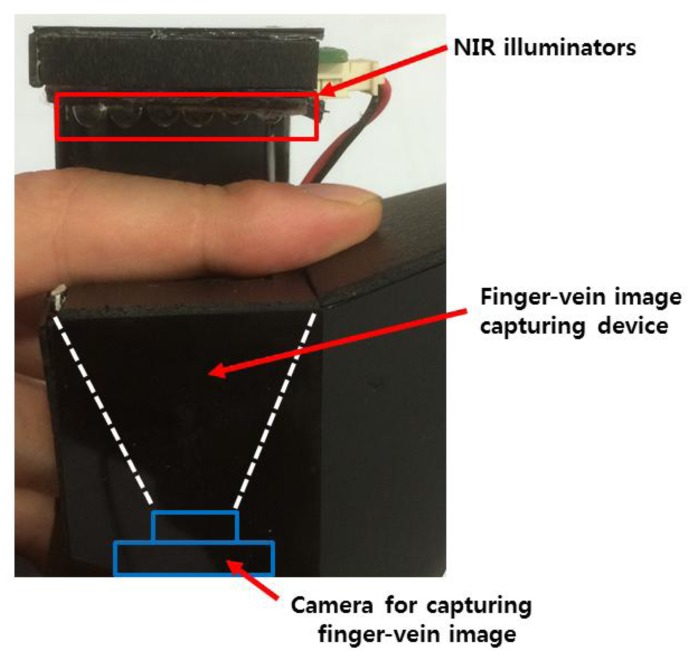
The image capture device made in the laboratory, which was used to obtain the finger-vein images in database I.

Two finger-vein image databases were used to verify the accuracy of finger-vein recognition using the proposed algorithm. Database I contained images captured by a device made in our laboratory, which comprised images of 33 people and the total number of images was 3,300 (33 people × 10 classes (10 fingers per person) × 10 images (10 per finger)). The image resolution was 640 × 480 pixels [6,17,20]. Figure 17 shows the finger-vein image-capture device used to produce database I, which comprised six NIR illuminators at 850 nm and a webcam. The width, height, and depth of the device were 43 mm, 100 mm, and 42 mm, respectively. The NIR illuminators were positioned on opposite sides of the camera for the following reasons. If the NIR illuminators were positioned at the sides of the finger, the camera could capture the finger-vein image while the finger is illuminated from the side. In this case, however, the uniformity of illumination would be degraded throughout the entire finger area. Thus, the image quality would be worse than that obtained when positioning the NIR illuminators above the finger's dorsal side, which was the position used for our device (Figure 17).

Database II comprised 3,816 finger-vein images (106 people × 6 classes (index, middle, and ring fingers of both hands) × 6 images (per finger)) and the image resolution was 320 × 240 pixels [21]. The equal error rate (EER) was measured to compare the accuracy of finger-vein recognition using the proposed quality enhancement method and a previous method. The EER is the error rate when the false acceptance rate (FAR) is most similar to the false rejection rate (FRR). The FAR indicates the error rate of non-enrolled people being incorrectly recognized as enrolled persons. The FRR denotes the error rate of enrolled people being rejected incorrectly as non-enrolled people [6,17,20]. For database I, the numbers of authentic and imposter matches were 14,850 (_10_C_2_ × 330) and 5,428,500 (_3300_C_2_ − 14,850), respectively. For database II, there were 9,540 (_6_C_2_ × 636) authentic matches and 7,269,480 (_3816_C_2_ − 9,540) imposter matches. In database I, the number of images in each class (finger) was 10. The number of images used for enrollment was changed in the 10 images, and therefore, the number of authentic matches with these 10 images was _10_C_2_. In addition, because the number of classes was 330 (33 people × 10 classes (10 fingers per person)), the total number of authentic comparisons was 14,850 (_10_C_2_ × 330). The imposter comparisons were performed using entire images, excluding the authentic comparisons, and therefore, the number of imposter comparisons was calculated as 5,428,500 (_3300_C_2_ − 14,850).

For database II, the number of image per class (finger) was 6. The number of images used for enrollment was changed in the six images, and therefore, the number of authentic matches with these six images was _6_C_2_. In addition, because the number of classes was 636 (106 people × 6 classes (index, middle, and ring fingers of both hands)), the total number of authentic comparisons was 9,540 (_6_C_2_ × 636). The imposter comparisons were performed using entire images, excluding the authentic comparisons, and therefore, the number of imposter comparisons was calculated as 7,269,480 (_3816_C_2_ – 9,540).

**Figure 18.**
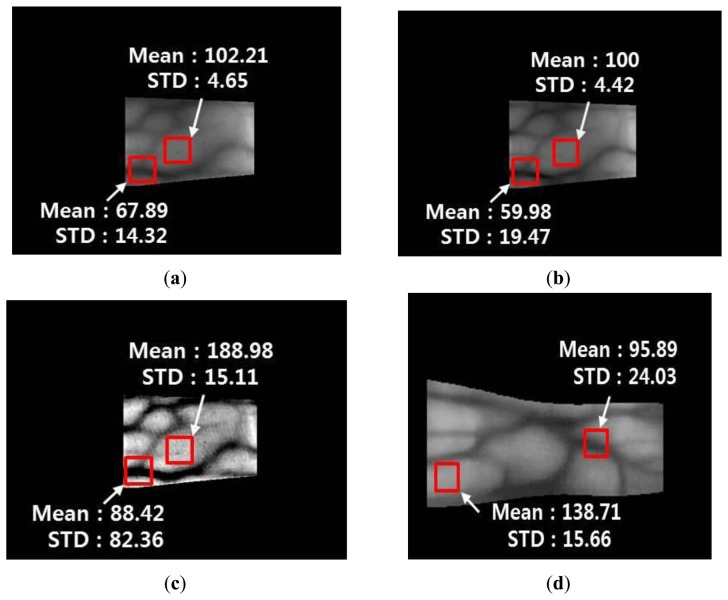
Comparison of the mean and STD values of the vein line and skin region using the proposed method with an image from database I: (**a**) original image of the detected finger boundaries; (**b**) Gabor filtered image; (**c**) Retinex filtered image with a sigma value of 20; and (**d**) image produced using the fuzzy-based fusion method with LOM and the Min rule (proposed method).

**Figure 19.**
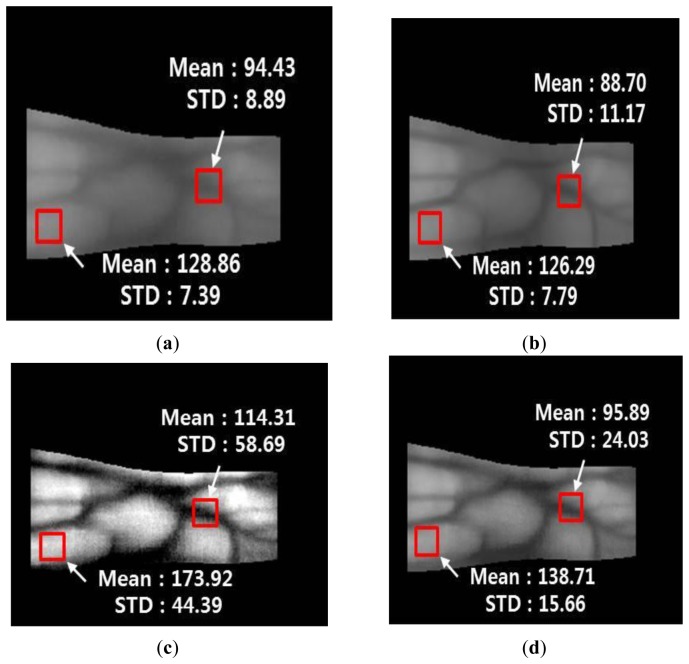
Comparison of the mean and STD values of a vein line and skin region using the proposed method for an image from database II: (**a**) original image of the detected finger boundaries; (**b**) Gabor filtered image; (**c**) Retinex filtered image with a sigma value of 20; and (**d**) image produced using the fuzzy-based fusion method with LOM and the Min rule (proposed method).

Figures 18 and 19 show the mean and STD values for vein lines and skin regions using the proposed method with images from database I and database II, respectively.

The mean values of the vein line regions of the Gabor filtered images are lower than those in the original images, whereas the STD values of the vein line regions of the Gabor filtered images are higher than those in the original images. In addition, the mean and STD values for the skin regions of the Gabor filtered images are similar to those of the original images. This indicates that the vein lines in the Gabor filtered images are more distinct than those in the skin regions. For the Retinex filtered image, both the mean and STD values of the vein line regions are higher than those in the original image. This shows that the contrast between the vein lines and skin regions in the Retinex filtered images is much higher than that in the original images. However, the Retinex filtered image has a problem because the noise is increased in the skin region, which is confirmed by the increase in the STD for the skin area of the Retinex filtered image compared with the original image. Figures 18d and 19d show enhanced images obtained with the fuzzy-based fusion method using LOM and the Min rule. A comparison of the means and STDs of the vein and skin areas in these images confirms that the proposed method reduces the noise in the skin region and enhances the contrast between the vein line and skin region.

### 3.1. Experimental Results with Database I

#### 3.1.1. Comparison of the Images Processed by Gabor Filtering, Retinex Filtering, and the Proposed Method

**Figure 20.**
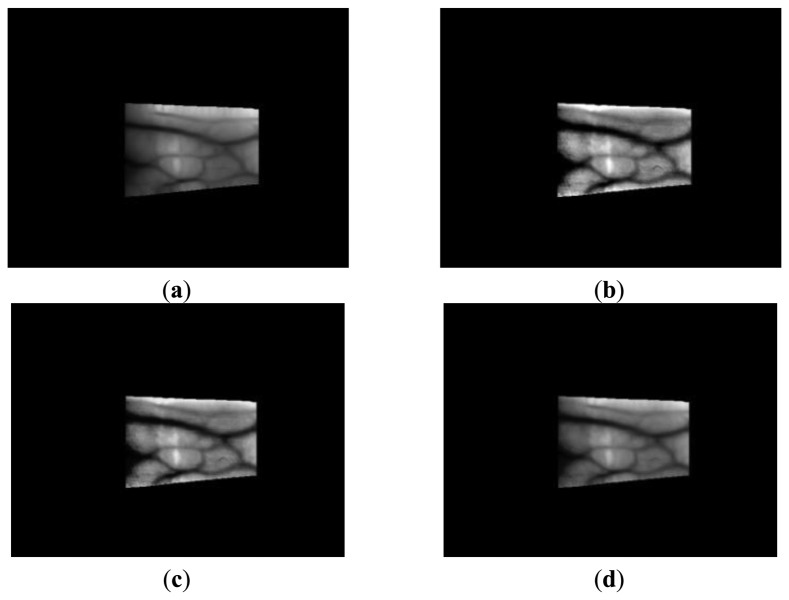

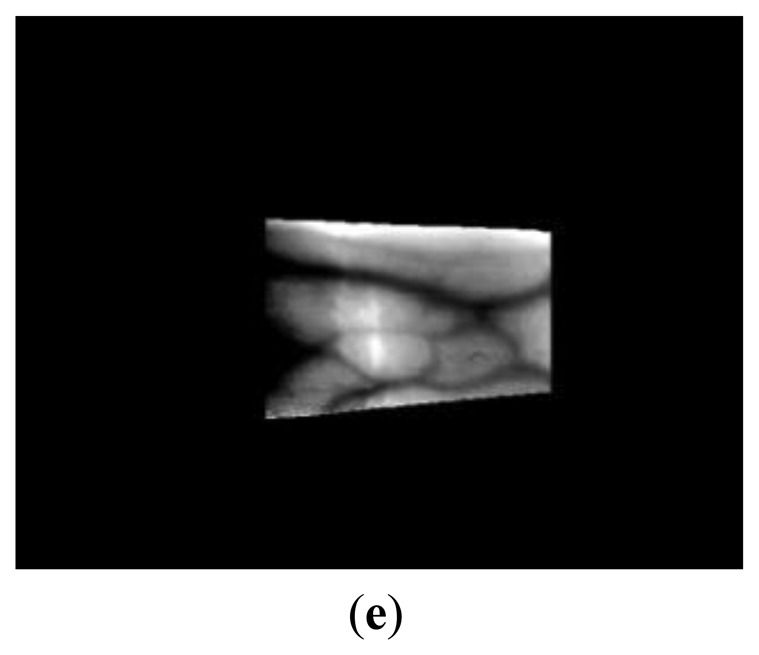
Enhanced images produced using the proposed method with four-directional Gabor filtering and Retinex filtering with a sigma value of 20 based on an image from database I: (**a**) Gabor filtered image; (**b**) Retinex filtered image with a sigma value of 20; (**c**) Fuzzy FOM based on the Min rule; (**d**) Fuzzy LOM based on the Min rule; and (**e**) Retinex filtered image with a sigma value of 50.

The proposed method was tested using a four-directional Gabor image and a Retinex image with sigma values of 10, 15, 20, 25, and 50 from database I. Figure 20 shows the enhanced images obtained by the fuzzy-based fusion of the Gabor filtered image and Retinex filtered image with a sigma value of 20. Figure 20c–d shows enhanced images obtained using the fuzzy-based fusion method, where the two images produced after Gabor filtering (Figure 20a) and Retinex filtering with a sigma value of 20 (Figure 20b) were combined with various defuzzification methods based on the Min rule. The images shown in Figures 20c–d were enhanced by reducing the noise and increasing the distinction between the vein line and skin region. In Figure 20d, the noise was reduced more in the skin region compared with the other enhanced images because the LOM defuzzification method selects the last output weight value (among all output values), which is relatively close to 1.

Therefore, the weight value of the Gabor filtered image is larger than that of the Retinex filtered image, as shown in Figure 11, and the noise is reduced by Gabor filtering. However, the contrast of the vein line was increased more in the images shown in Figure 20c compared with the other enhanced images by FOM defuzzification because the FOM method selects the first output weight value (among all output values), which is relatively close to 0. Therefore, the weight value for a Retinex filtered image is larger than that of a Gabor filtered image, as shown in Figure 11, and the contrast of the vein line is increased. The sigma value of Retinex filtering determines the size of the Gaussian filter. It was confirmed that the noise was reduced in a Retinex filtered image with a sigma value of 50 (Figure 20e) compared with a sigma value of 20 (Figure 20b). In addition, the image quality of the image produced was increased compared with the original and the Gabor and Retinex filtered images, as shown in Figure 20. The lowest EER was obtained with Fuzzy LOM based on the Min rule and we illustrate this in Figure 20d. The highest recognition accuracy with database I was obtained using the LBP method and Retinex filtering with a sigma value of 20, as shown in the following tables and figures, and therefore, we illustrate the image produced by Retinex filtering with a sigma value of 20.

#### 3.1.2. Comparison of the Accuracy of Finger-Vein Recognition Using the LBP Method

As shown in Table 4, the recognition accuracy of the proposed method based on a four-directional Gabor filtered image and Retinex filtered image with sigma values of 10, 15, 20, 25, and 50 was compared using images from database I. The recognition accuracy is expressed in terms of EER based on an LBP operator. The recognition accuracy of Retinex filtered images with sigma values of 15–50 were enhanced by reducing the noise in the skin region compared with the Retinex filtered image with a sigma value of 10.

**Figure 21.**
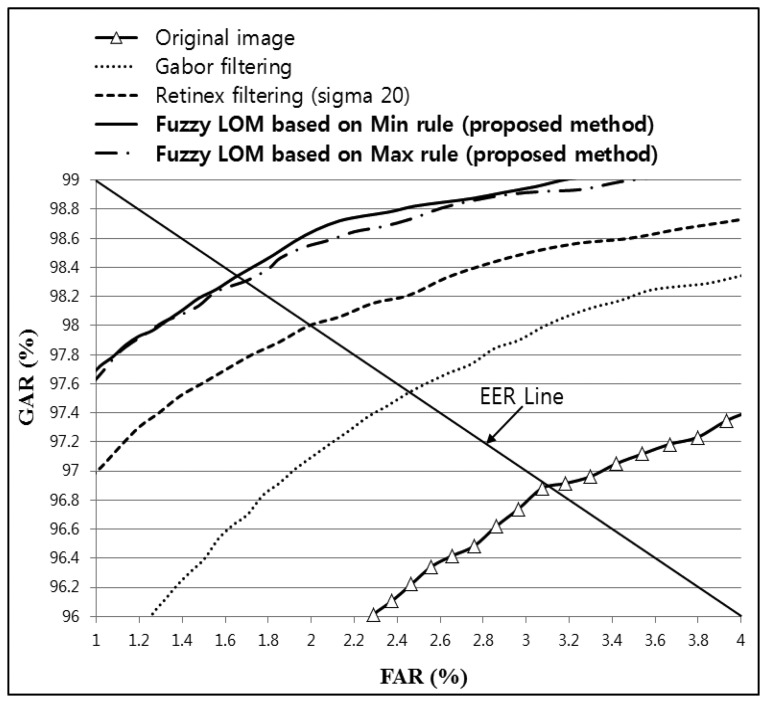
ROC curves obtained using the proposed method with Gabor filtering and Retinex filtering, with a sigma value of 20, and a LBP operator for images from database I.

As shown in Table 4; the lowest EER (1.6561%) was obtained by combining Gabor and Retinex filtered images with a sigma value of 20 and using fuzzy-based fusion (Min + LOM method); which was lower than that for the original; Gabor; or Retinex filtered images. In addition; the recognition accuracy with the proposed method was increased with all sigma values for Retinex images compared with the original; Gabor; or Retinex filtered images; as shown in Table 4. Figure 21 shows the receiver operational characteristic (ROC) curves for the proposed method using a Gabor filtered image and a Retinex filtered image with a sigma value of 20; which demonstrates that the proposed method outperformed other methods. The genuine acceptance rate (GAR) was calculated as 100 − FRR (%).

#### 3.1.3. Comparison of the Finger-vein Recognition Accuracy Using Daubechies Wavelet Method

**Figure 22.**
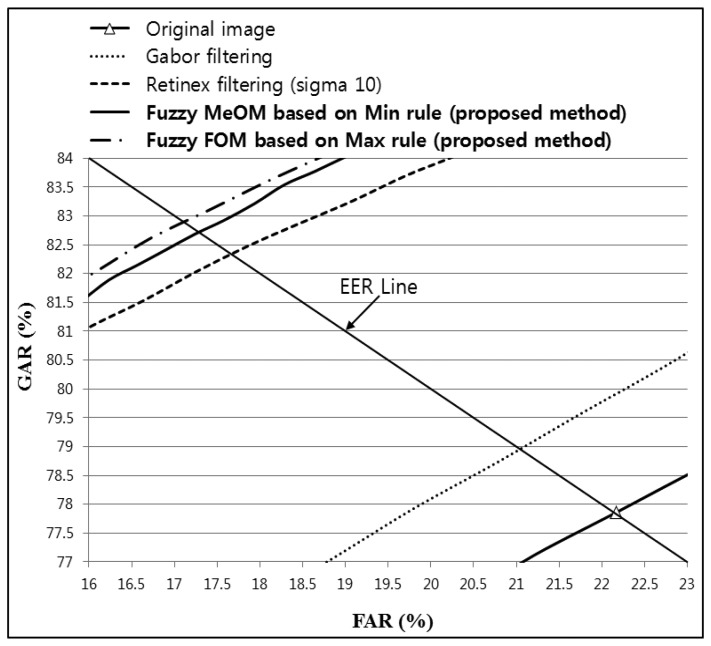
ROC curves obtained using the proposed method with Gabor filtering and Retinex filtering, with a sigma value of 10, and a Daubechies wavelet for images from database I.

To demonstrate that the proposed method can enhance the recognition accuracy regardless of the type of recognition algorithm used, additional experiments were conducted using finger-vein recognition based on a Daubechies wavelet. Table 5 shows the finger-vein recognition accuracy for an original image, Gabor filtering, Retinex filtering, and the proposed method based on images from database I. As shown in Table 5, fuzzy-based fusion using the proposed method increased the recognition accuracy compared with the original image and both Gabor and Retinex filtering. In addition, the accuracy comparison shown in Table 5 indicates that the lowest EER obtained was 17.1340%, which was achieved by the proposed fuzzy-based fusion method with Gabor and Retinex filtering (sigma value of 10) using FOM and the Max rule, as shown in Table 5 and Figure 22.

#### 3.1.4. Comparison of Finger-vein Recognition Accuracy Using a Haar Wavelet Method

**Figure 23.**
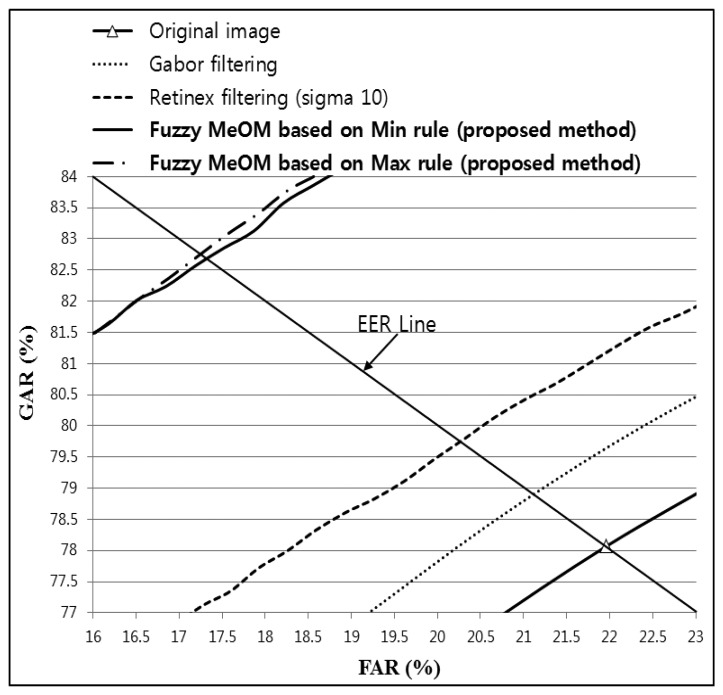
ROC curves obtained using the proposed method with Gabor filtering and Retinex filtering, with a sigma value of 10, and a Haar wavelet for images from database I.

To demonstrate that the proposed method can enhance the recognition accuracy regardless of the type of recognition algorithm used, additional experiments were conducted using finger-vein recognition based on a Haar wavelet. Table 6 shows the accuracy of finger-vein recognition for the original image, Gabor filtering, Retinex filtering, and the proposed method using images from database I. Table 6 confirms that fuzzy-based fusion using the proposed method increased the recognition accuracy compared with the original image and both Gabor and Retinex filtering. A comparison of the accuracies in Table 6 shows that the lowest EER was 17.2472%, which was achieved using the proposed fuzzy-based fusion of Gabor and Retinex filtering (sigma value of 10) based on MeOM and the Max rule, as shown in Table 6 and Figure 23.

### 3.2. Experimental Results with Database II

#### 3.2.1. Comparison of Images Processed Using Gabor Filtering, Retinex Filtering, and the Proposed Method

**Figure 24.**
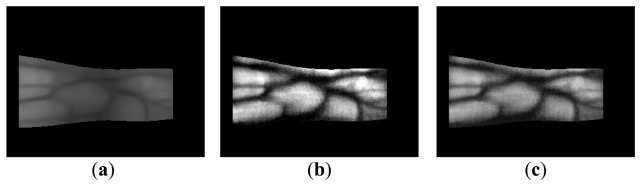
Enhanced images obtained using the proposed method with four-directional Gabor filtering and Retinex filtering with a sigma value of 15 using images from database II: (**a**) Gabor filtered image; (**b**) Retinex filtered image with a sigma value of 15; and (**c**) Fuzzy FOM based on the Max rule.

To demonstrate the increased recognition accuracy with the proposed method regardless of the type of database used, additional experiments were conducted with images from database II. Figure 24 shows the images produced with the proposed method using Gabor filtered images and Retinex filtered images with sigma values of 15 for images from database II.

**Figure 25.**
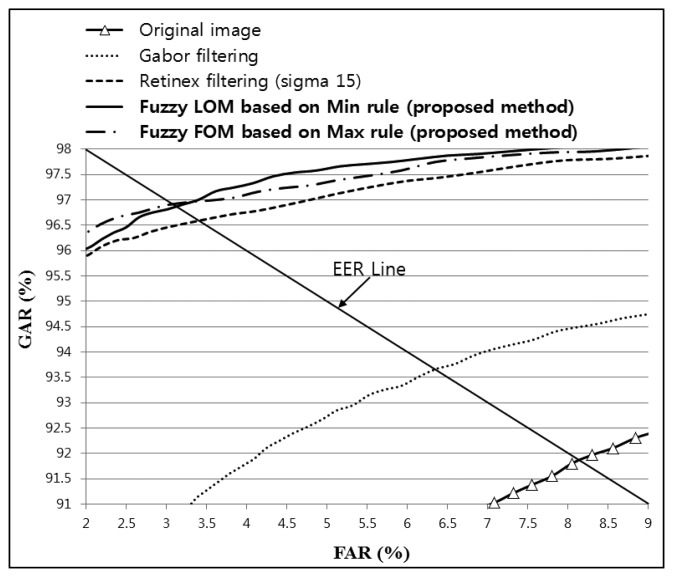
ROC curves obtained using the proposed method with Gabor filtering and Retinex filtering, with a sigma value of 15, and a LBP operator for images from database II.

The images produced using the proposed method were enhanced compared with the original and the Gabor and Retinex filtered images, as shown in Figure 24. The lowest EER was obtained with Fuzzy FOM based on the Max rule, as shown in Table 7; thus, we illustrate this case in Figure 24c. In addition, the highest recognition accuracy obtained by the LBP method was with Retinex filtering and a sigma value of 15 for images from database II, as shown in Table 7 and Figure 25, and therefore, we only show an image produced by Retinex filtering with a sigma value of 15.

#### 3.2.2. Comparison of Finger-vein Recognition Accuracy Using the LBP Method

As shown in Table 7, the recognition accuracy of the proposed method based on a four-directional Gabor filtered image and Retinex filtered images was compared with sigma values of 10, 15, 20, 25, and 50 using images from database II. Table 7 confirms that fuzzy-based fusion using the proposed method increased the recognition accuracy compared with the original image and both Gabor and Retinex filtering. In addition, the comparison of the accuracy shown in Table 7 demonstrates that the lowest EER was 3.0846%, which was achieved using the proposed fuzzy-based fusion of Gabor and Retinex filtering (sigma value of 15) based on FOM and the Max rule, as shown in Table 7 and Figure 25. The EER was higher with database II than that with database I (Table 4) because the image resolution of database II (320 × 240 pixels) was lower than that of database I (640 × 480 pixels). In addition, the noise and blurring were higher in images from database II compared with those from database I, which reduced the distinctiveness of the vein line.

Two types of matching schemes are used in biometrics: identification and verification. In identification methods, one input data item is matched with multiple enrolled data without supplementary ID information such as usernames (1 to N matching). In verification methods, one input data item is matched with only one enrolled item, which is determined using additional ID information (1 to 1 matching). The recognition accuracy is usually measured in terms of rank during identification (rank 1, rank 10, *etc.*), whereas it is measured in terms of the EER and ROC curves during verification. Our study aimed to develop a finger-vein verification system, so the accuracy was measured in terms of the EER and ROC curves.

### 3.3. Processing Time of the Proposed Method

Finally, the processing time of the proposed method was measured on a desktop computer with an Intel Core i7 processor at 3.33 GHz and 4 GB of RAM, as shown in Table 8. For database I, we used LOM and the Min rule because the accuracy of finger-vein recognition using LOM and the Min rule with the LBP method was higher than that with other methods, as shown in Table 4. In addition, we used FOM and the Max rule for database II because the accuracy of finger-vein recognition using FOM and the Max rule with the LBP method was higher than that with other methods, as shown in Table 7.

As shown in Table 8, the total processing times for each image from databases I and II were 523.733 ms and 209.233 ms, respectively, which shows that our method can be used as a real-time finger-vein recognition system. The processing time for each image from database II was less than that for images from database I because the image size used in database II (320 × 240) was smaller than that in database I (640 × 480).

In our method, image enhancement is achieved using a combination of both Gabor and Retinex filtering based on a fuzzy system. The fuzzy system can be designed heuristically without a training procedure (which is required for neural network systems) to obtain the optimal weights for the combination of Gabor filtering and Retinex filtering. Therefore, this fuzzy-based system has the advantage that it does not need to be redesigned for different types of finger-vein databases, whereas a neural network-based system must be trained to suit specific databases.

In this study, we demonstrated an image enhancement method for finger-vein recognition but our system does not have the function for template protection. However, a previous study [5] proposed a cancellable and non-invertible finger-vein recognition system based on bio-hashing, fuzzy commitment and fuzzy vault sketches, and a fusion method. Our finger-vein features based on Haar and Daubechies wavelets are real values, similar to those used by their method based on Gabor filter and linear discriminant analysis (LDA) [5], thus our finger-vein features can be transformed into cancellable and non-invertible features using their method [5]. The finger-vein features used by the LBP operator are binary bits, but they can also be transformed into cancellable and non-invertible features using their method if the binary bit features are first represented as real values via additional processing by clustering, *etc.* [28]. In future research, we plan to implement this method for template protection [5] in our finger-vein recognition system, which will make the template of our system cancellable and more secure.

## 4. Conclusions

In this study, we developed a fuzzy-based image fusion algorithm for enhancing the quality of a finger-vein images, which can be degraded by various factors such as the light scattering from the skin and the finger thickness. Gabor filters in four directions and Retinex filtering were used to amplify the local and global features of the vein lines in the input image. The optimal weights for the fuzzy-based fusion method were determined using the mean and STD values in the local windows of the images produced by Gabor filtering and Retinex filtering, which were employed as the inputs for the fuzzy rule and fuzzy membership function. Based on the optimal weights obtained, finger-vein image enhancement was achieved by combining the Gabor and Retinex filtered images. The experimental results showed that the finger-vein recognition accuracy was enhanced using the proposed method compared with other methods based on images from two finger-vein databases. Further, this was confirmed using various finger-vein recognition algorithms such as LBP and DWT based on Daubechies and Haar wavelets. In future research, we plan to apply the proposed method to hand vein or palm vein images. In addition, we will test the possibility of applying our method to other biometric modalities such as face, iris, and fingerprint images.

## Figures and Tables

**Table 1.** Comparison of the proposed method and previous methods.

**Category**	**Restoration-Based Method**	**Non-Restoration-Based Method**		
		**Single Image-Based Method**		**Multiple Image-Based Method**
Method	Restoration-based on optical blur caused by camera lens, and skin scattering blur by the skin layer [7].Restoration method based on de-hazing and skin scattering blur [8].	Method using gray-level grouping and a circular Gabor filter for contrast and image enhancement [10].Method using edge-preserving and elliptical high-pass filters, and histogram equalization [11].Fuzzy-based multi-threshold algorithm [12].Method using multi-channel Gabor and image reconstruction [13].	Method using optimal Gabor filter based on the direction and thickness of the vein line [6].Method using coarse vein-width variation field and primary orientation field [14].Method using vein line tracking and adaptive Gabor filtering [15].	Combination of Gabor and Retinex filters based on fuzzy theory **(proposed method)**
Strength	Various vein-patterns can be distinguished by removing blur effects.	The contrast between vein patterns and skin regions is increased.The proposed method is straightforward in terms of image enhancement.	Information related to the orientation and width of the vein line is considered during image enhancement.	Local and global features of a finger-vein are considered.The performance is not affected by detection errors in the orientation and thickness of a vein line.
Weakness	The direction and width of the vein are not considered during restoration. The performance can be affected by the detection of the scattering parameter.No enhancement of the recognition accuracy was demonstrated.	The direction and width of the vein are not considered. No enhancement of the recognition accuracy was demonstrated.	Detection errors in the orientation and thickness of a vein line can affect the performance.	The processing time is increased by the use of both Gabor and Retinex filters.

**Table 2.** Fuzzy rules based on the characteristics of the Gabor and Retinex filtered images.

**Input 1 (*u****_1_***) of** **Gabor Image**	**Input 2 (*std****_1_***) of** **Gabor Image**	**Input 3 (*u****_2_***) of** **Retinex Image**	**Input 4 (*std****_2_***) of** **Retinex Image**	**Output (*w*) of** **Gabor Image**
L	L	L	L	M
L	L	L	H	L
L	L	H	L	M
L	L	H	H	L
L	H	L	L	H
L	H	L	H	M
L	H	H	L	H
L	H	H	H	H
H	L	L	L	M
H	L	L	H	L
H	L	H	L	M
H	L	H	H	L
H	H	L	L	H
H	H	L	H	L
H	H	H	L	H
H	H	H	H	M

**Table 3.** Illustrations of 16 combination pairs of output values for four membership functions.

***Pair Index***	***Output of f****_1_****(˙)***	***Output of f****_2_****(˙)***	***Output of f****_3_****(˙)***	***Output of f****_4_****(˙)***	***Min*** ***Rule***	***Max*** ***Rule***
1	0.39 (L)	0.55 (L)	0.67 (L)	0.27 (L)	0.27 (M)	0.67 (M)
2	0.39 (L)	0.55 (L)	0.67 (L)	0.73 (H)	0.39 (L)	0.73 (L)
3	0.39 (L)	0.55 (L)	0.33 (H)	0.27 (L)	0.27 (M)	0.55 (M)
4	0.39 (L)	0.55 (L)	0.33 (H)	0.73 (H)	0.33 (L)	0.73 (L)
5	0.39 (L)	0.45 (H)	0.67 (L)	0.27 (L)	0.27 (H)	0.67 (H)
6	0.39 (L)	0.45 (H)	0.67 (L)	0.73 (H)	0.39 (M)	0.73 (M)
7	0.39 (L)	0.45 (H)	0.33 (H)	0.27 (L)	0.27 (H)	0.45 (H)
8	0.39 (L)	0.45 (H)	0.33 (H)	0.73 (H)	0.33 (H)	0.73 (H)
9	0.61 (H)	0.55 (L)	0.67 (L)	0.27 (L)	0.27 (M)	0.67 (M)
10	0.61 (H)	0.55 (L)	0.67 (L)	0.73 (H)	0.55 (L)	0.73 (L)
11	0.61 (H)	0.55 (L)	0.33 (H)	0.27 (L)	0.27 (M)	0.61 (M)
12	0.61 (H)	0.55 (L)	0.33 (H)	0.73 (H)	0.33 (L)	0.73 (L)
13	0.61 (H)	0.45 (H)	0.67 (L)	0.27 (L)	0.27 (H)	0.67 (H)
14	0.61 (H)	0.45 (H)	0.67 (L)	0.73 (H)	0.45 (L)	0.73 (L)
15	0.61 (H)	0.45 (H)	0.33 (H)	0.27 (L)	0.27 (H)	0.61 (H)
16	0.61 (H)	0.45 (H)	0.33 (H)	0.73 (H)	0.33 (M)	0.73 (M)

**Table 4.** Recognition accuracy using the proposed method with Gabor filtering and Retinex filtering, with various sigma values, and a LBP operator in terms of EER for images from database I (unit: %).

**Method**		**Sigma Value**				

		**10**	**15**	**20**	**25**	**50**
Original image		3.0957				
Gabor filtering		2.4564				
Retinex filtering		3.1163	2.2406	1.9943	1.9628	2.2665
Fuzzy Min rule (Gabor + Retinex)	FOM	2.8444	2.0907	1.8917	1.8809	2.2875
	**LOM**	2.0971	1.6714	**1.6561**	1.7312	2.1336
	MOM	2.5221	1.9813	1.7711	1.7549	2.2216
	MeOM	2.5568	1.8881	1.7948	1.7513	2.2414
	COG	2.5501	1.8498	1.7747	1.7803	2.1826
Fuzzy Max rule (Gabor + Retinex)	FOM	2.6480	1.9548	1.8337	1.8131	2.2115
	**LOM**	2.2569	1.7729	**1.6718**	1.7184	2.1920
	MOM	2.5300	1.9000	1.7551	1.7527	2.2166
	MeOM	2.3536	1.8121	1.7286	1.7011	2.1622
	COG	2.5560	1.8582	1.7749	1.7681	2.1911

**Table 5.** Recognition accuracy of the proposed method with Gabor filtering and Retinex filtering, with various sigma values, and a Daubechies wavelet in terms of EER for images from database I (unit: %).

**Method**		**Sigma value**				

		**10**	**15**	**20**	**25**	**50**
Original image		22.1564				
Gabor filtering		21.0246				
Retinex filtering		17.6892	19.1360	20.4271	21.2442	22.6522
Fuzzy Min rule (Gabor + Retinex)	FOM	17.6550	18.9607	19.9225	20.6797	22.0395
	LOM	18.2462	19.1647	19.8896	20.2432	20.8612
	MOM	17.2971	18.5995	19.6813	20.4106	21.3814
	**MeOM**	**17.2776**	18.5480	19.7489	20.3397	21.4183
	COG	17.2790	18.6564	19.6560	20.4020	21.4573
Fuzzy Max rule Gabor + Retinex)	**FOM**	**17.1340**	18.5820	19.7045	20.5765	21.7044
	LOM	17.6858	18.6977	19.6775	20.1349	21.1535
	MOM	17.2861	18.5965	19.6664	20.4055	21.3796
	MeOM	17.1869	18.5296	19.6425	20.3179	21.4062
	COG	17.2500	18.6302	19.7060	20.3905	21.4376

**Table 6.** Recognition accuracy of the proposed method with Gabor filtering and Retinex filtering, with various sigma values, and a Haar wavelet in terms of the EER for images from database I (unit: %).

**Method**		**Sigma Value**				

		**10**	**15**	**20**	**25**	**50**
Original image		21.9457				
Gabor filtering		21.0902				
Retinex filtering		20.2605	19.9946	20.1749	20.2424	21.1762
Fuzzy Min rule (Gabor + Retinex)	FOM	18.2522	18.5999	19.2453	19.7527	20.6428
	LOM	18.1611	18.3407	18.7428	19.0482	19.9215
	MOM	17.3471	18.1422	18.7486	19.2664	20.2761
	**MeOM**	**17.3105**	18.0367	18.6332	19.2486	20.2888
	COG	17.3556	18.0572	18.7200	19.3121	20.2168
Fuzzy Max rule (Gabor + Retinex)	FOM	17.6965	18.2328	18.7112	19.1041	20.1956
	LOM	17.5980	18.4322	19.1212	19.5938	20.3843
	MOM	17.3273	18.0621	18.7441	19.2559	20.2573
	**MeOM**	**17.2472**	18.0280	18.7272	19.2473	20.3109
	COG	17.3116	18.1001	18.7230	19.3365	20.1999

**Table 7.** Recognition accuracy of the proposed method with Gabor filtering and Retinex filtering using various sigma values and a LBP operator in terms of the EER for images from database II (unit: %).

**Method**		**Sigma Value**				

		**10**	**15**	**20**	**25**	**50**
Original image		8.1231				
Gabor filtering		6.3478				
Retinex filtering		3.6255	3.3674	3.2498	3.6085	5.4432
Fuzzy Min rule (Gabor + Retinex)	FOM	3.4471	3.2076	3.2161	3.5351	5.2578
	**LOM**	3.1880	**3.1467**	3.3996	3.7709	5.4413
	MOM	3.3134	3.1503	3.2830	3.5735	5.3588
	MeOM	3.3339	3.1536	3.3138	3.5187	5.2382
	COG	3.2849	3.1951	3.2677	3.5347	5.3445
Fuzzy Max rule (Gabor + Retinex)	**FOM**	3.2984	**3.0846**	3.1019	3.4455	5.1466
	LOM	3.2029	3.1909	3.3226	3.7220	5.4485
	MOM	3.3111	3.1406	3.2919	3.5345	5.2446
	MeOM	3.3391	3.1480	3.2722	3.5497	5.2568
	COG	3.2609	3.1820	3.2468	3.5004	5.3041

**Table 8.** Processing time for each sub-module of the proposed method per image.

**Sub-Modules**		**Processing Time (ms)**	

		**Database I**	**Database II**
Image preprocessing	Finger region detection	10.3	5.3
Image enhancement	Four-directional Gabor filtering	83.1	51.5
	Retinex filtering	380.4	124.8
	Image fusion by fuzzy logic	36.1	13.9
Feature extraction and matching	Size normalization	0.3	0.2
	Finger-vein code extraction by the LBP operator and HD calculation	13.533	13.533
Total		523.733	209.233
